# Right forearm wound and soft tissue abscess complicated with sepsis due to Streptococcus suis: a case report

**DOI:** 10.3389/fmed.2026.1866162

**Published:** 2026-07-10

**Authors:** Hua Ding, Chao Lei, Desheng Liu, Qing Tian

**Affiliations:** 1 Hospital of Chengdu University of Traditional Chinese Medicine, Chengdu, China; 2 Tongren Hospital of Traditional Chinese Medicine, Tongren, China; 3 Sichuan Province Orthopedic Hospital, Chengdu, Sichuan, China

**Keywords:** case report, spesis shock, *Streptococcus suis*, vancomycin-loaded bone cement, wound abscess

## Abstract

**Background:**

*Streptococcus suis (S. suis)* is an opportunistic zoonotic bacterial pathogen that is typically transmitted to humans through injured skin or mucous membranes, or through consumption of undercooked contaminated pork. It primarily causes meningitis, septicemia, arthritis, and endocarditis. Reports describing wound infection as the predominant clinical manifestation of *S. suis* infection are rare. We report a case of *S. suis* wound infection complicated by sepsis.

**Case presentation:**

A 49-year-old woman presented to the emergency department with swelling and pain in her right forearm following wound debridement. According to her history, after wound debridement, she consumed pork soup at a local eatery, where the serving container may have been in contact with contaminated with raw pork. Both blood and wound pus cultures indicated infection with *S. suis*. Vancomycin and metronidazole were used to control the infection, while plasma transfusion was administered to improve coagulation. Multiple debridements, skin grafting, and vacuum sealing drainage (VSD) were performed. At the 1-year follow-up, the patient achieved a satisfactory recovery and remained free of complications.

**Conclusion:**

*S. suis* infections are commonly associated with individuals who consume raw or undercooked pork and occupational exposure in high-risk groups, including farm workers and butchers. However, trauma-associated infections are more commonly attributed to *Staphylococcus aureus*. This report draws attention to a possible association between wound infections in trauma patients and *S. suis* exposure, suggesting that this factor may be considered during clinical evaluation. Therefore, timely wound secretion and blood cultures play a critical role in accurate diagnosis and effective treatment.

## 1 Introduction

*S. suis* is an emerging zoonotic pathogen, increasingly recognised for its public health impact and its burden on the swine industry. The highest global incidence is reported in Southeast Asia of *S. suis* infections, particularly in Thailand and Vietnam, where cultural practices of consuming raw pork products significantly contribute to the prevalence (1). In China, the majority of cases were reported during the outbreaks in 1998 and 2005 (2). During non-epidemic periods, the incidence is sporadic and rarely encountered in clinical practice. Meningitis and sepsis are the most common clinical manifestations in human infections (3). According to the literature, human infections caused by serotype 2 account for 97%, with a small number attributed to serotypes 1, 1/2, and 4 (4, 5). Wound infections complicated by sepsis caused by *S. suis* are rare. This article reports the clinical course and management of a case treated at Tongren Hospital of Traditional Chinese Medicine, and reviews previously reported cases to enhance understanding of this condition.

## 2 Case report

A 49-year-old female presented to our hospital witha 36-h history of swelling and pain in her right forearm following trauma. The patient, an office worker living in an urban area, sustained the injury at home on a door frame. She then went to a local hospital, where the wound was debrided and sutured. No antibiotics were administered at that time. She reported no direct contact with pigs or raw pork. After wound management, she went to a restaurant and consumed pork soup. On the evening of the same day, she felt swelling and pain at the wound site but did not seek further medical attention. The pain progressively worsened and became intolerable the following day, so she presented to our hospital for further evaluation and management.

**Figure 1:**
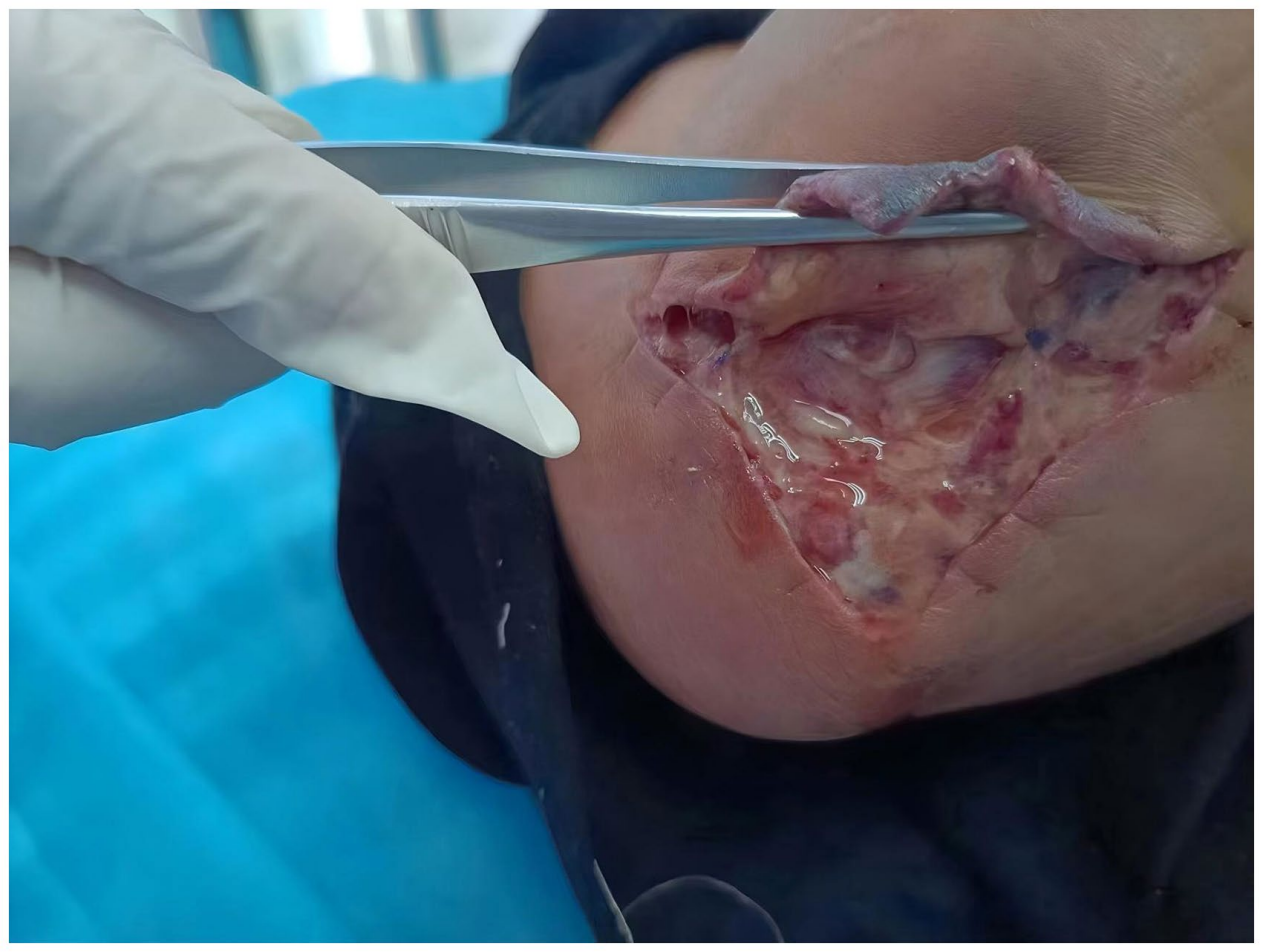
*S. suis* forearm wound abscess. Appearance at the time of suture removal upon admission in a 58-year-old female patient.

33 h after wound debridement and suturing, the patient presented to our emergency department. On admission, the vital signs were as follows: body temperature, 36.6 °C; blood pressure, 70/40 mmHg; heart rate, 110 beats/min; respiratory rate, 23 breaths/min; and glucose level, 4.5 mmol/L. Subsequent blood tests revealed: white blood cell count 5.68 × 10^9/L, neutrophil percentage 88.2%, absolute neutrophil count 5 × 10^9/L platelet count 104.0 × 10^9/L, C-reactive protein level 115 mg/L, procalcitonin 7.2 ng/mL, red blood cells 3.95 × 10^12/L, hemoglobin level 109 g/L, B-type natriuretic peptide level 2037.7 pg./mL, blood potassium level 3.36 mmol/L, D-dimerlevel0.3 mg/L, Alanine aminotransferase was 36.9 U/L, aspartate aminotransferase was 45.0 U/L, creatinine was 127.04 μmol/L, and lactate was 3.2 mmol/L. She fulfilled the Sepsis-3 definition of septic shock, characterized by a confirmed infection (positive blood and wound cultures), refractory hypotension (70/40 mmHg) requiring dopamine infusion to maintain a mean arterial pressure ≥65 mmHg, and hyperlactatemia (3.2 mmol/L). 2 h later, she was transferred to the intensive care unit (ICU) for further management. On ICU admission, she reported persistent dizziness and palpitations without hearing impairment. She was conscious but lethargic (GCS score, 15). Vital signs were as follows: body temperature, 36.6 °C; Blood pressure was 86/51 mmHg under dopamine infusion. The heart rate was 121 beats/min with a regular rhythm and weak, thready peripheral pulses. No cardiac murmurs were detected; respiratory rate, 25 breaths/min; and capillary refill time >3 s. Pulmonary auscultation was clear, with no crackles or wheezes. The abdomen was soft and non-tender, with no hepatosplenomegaly or costovertebral angle tenderness. No tenderness was elicited over the spinal column. Neurological examination was unremarkable, with a negative Babinski sign and normal deep tendon reflexes. Meningeal signs were absent. Surgical history included one prior cesarean section. There was no history of hypertension, diabetes mellitus, or coronary artery disease. Occasional light alcohol consumption was reported, and there was no smoking history. HIV infection, tuberculosis, and hepatitis B infection were denied. No known drug or food allergies were reported. Physical examination revealed a wound dressing saturated with yellowish exudate and mild black discoloration at the incision margins. Marked swelling involving the right forearm, palm, and elbow was associated with tension blisters. The overlying skin was erythematous and warm (Figure 1).

**Figure 2:**
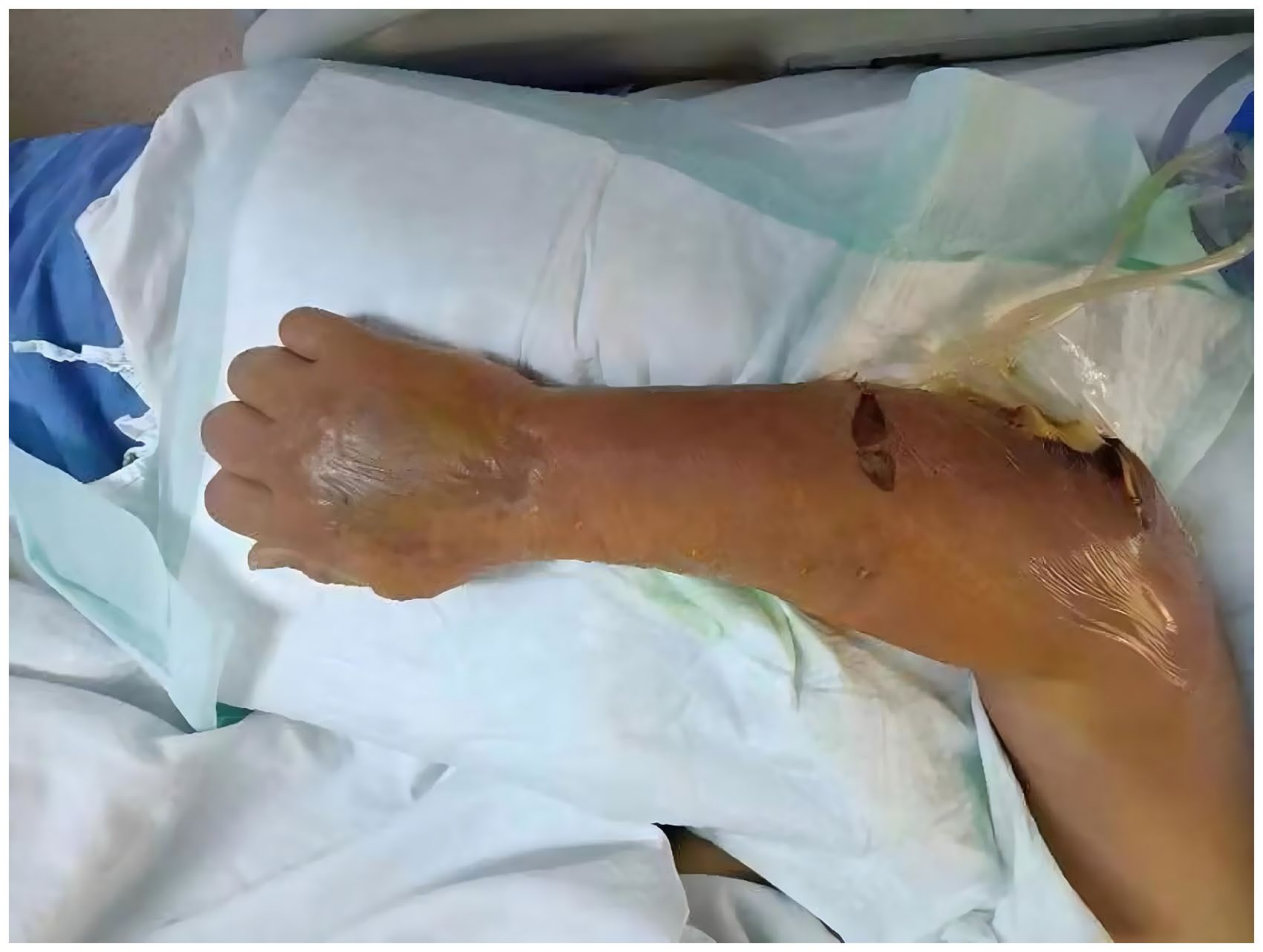
*S. suis* skin and soft tissue abscess. Forearm wound after initial debridement and placement of vacuum sealing drainage (VSD) system.

Initially, we suspected *Staphylococcus aureus* or *Streptococcus pyogenes* (group A streptococcus, GAS) infection. Piperacillin-tazobactam 4.5 g every 12 h and metronidazole 1 g once daily were administered for anti-infective treatment. Meanwhile, dopamine was continuously infused as a vasopressor at >10 μg/kg/min. Intravenous Xuebijing (100 mL twice daily for 7 days) and ulinastatin (200,000 U twice daily for 10 days) were administered for anti-inflammatory purposes, along with other supportive therapy. Additionally, wound management included surgical debridement and negative pressure therapy with vacuum-sealed drainage (VSD) (Figure 2). Concurrently, exudate and blood samples were collected for microbiological analysis to facilitate the selection of appropriate antimicrobials.

The patient’s left forearm oedema progressed despite aggressive antimicrobial treatment. The patient’s complete blood count showed a white blood cell count of 13.36 × 10^9/L, a neutrophil percentage of 87.2%, a neutrophil count of 11.8 × 10^9/L, and a procalcitonin level of 2.88 ng/mL. On hospital day 3 (i.e., 3 days after specimen collection), *S. suis* was identified from wound exudate using the XinKe bacterial identification and antimicrobial susceptibility analyzer (Scenker Biological Technology Co., Ltd., China), which employs colorimetric and turbidimetric principles. The isolate was classified as *S. suis* type 1 (hereafter referred to as biotype I). During hospitalization, the same pathogen was repeatedly isolated from two blood cultures and four wound exudate cultures, with antimicrobial susceptibility profiles demonstrating complete concordance across all isolates. Molecular confirmation could not be performed owing to laboratory limitations. Minimum inhibitory concentration (MIC) values for key antibiotics, interpreted according to the Clinical and Laboratory Standards Institute (CLSI) M100 guidelines (30th edition), are summarized in Table 1.

**Table 1:** Minimum inhibitory concentrations (MICs) and susceptibility interpretation of the *S. suis* isolate.

Antibiotic	MIC (μg/mL)	Interpretation
Penicillin	4	Resistant
Ampicillin	0.5	Intermediate
Ceftriaxone	≤0.5	Susceptible
Vancomycin	≤0.5	Susceptible
Meropenem	≤0.25	Susceptible
Clindamycin	≤0.12	Susceptible
Levofloxacin	≤2	Susceptible
Linezolid	≤2	Susceptible
Erythromycin	≤0.12	Susceptible

MIC, minimum inhibitory concentration; CLSI, Clinical and laboratory standards institute.

Accordingly, the antimicrobial regimen was changed to intravenous vancomycin (1 g every 12 h for 14 days) plus intravenous metronidazole (500 mg every 8 h for 14 days). Metronidazole was added because traumatic wound infections are frequently polymicrobial, involving both aerobic and anaerobic bacteria (6). *Streptococcus species* commonly coexist with anaerobes in soft-tissue infections (7). Although the *S. suis* isolate was not specifically tested for metronidazole susceptibility, anaerobic coverage was considered clinically appropriate. The subsequent rapid clinical and laboratory improvement after the regimen was changed to vancomycin plus metronidazole supports the clinical rationale for this decision (Table 2).

**Table 2:** Blood test results and surgical treatment methods during the first 5 days of hospitalization in a patient with *S. suis* skin and soft tissue abscess complicated by septic shock.

Date	WBC (×10^9^ /L)	ANC (×10^9^ /L)	NEUT%	CRP (mg/L)	sCr (μmol/L)	PCT (ng/mL)	Antibiotics	Surgical procedures
Day 1 of admission	5.68	5.00	88.20	115.08	127.04	7.20	Piperacillin–tazobactam	Debridement + VSD
Day 2 of admission	3.34	3.10	93.20	113.97	117.34	7.34	Piperacillin–tazobactam	Dressing change
Day 3 of admission	7.87	7.30	92.70	108.23	110.54	2.88	Piperacillin–tazobactam	Dressing change
Day 4 of admission	13.36	11.8	88.20	98.70	85.20	1.63	Vancomycin + metronidazole	forearm fasciotomy + antibiotic-loaded bone cement
Day 5 of admission	14.41	11.40	78.90	43.83	83.45	0.67	Vancomycin + metronidazole	Dressing change

WBC, white blood cell; ANC, absolute neutrophil count; NEUT, neutrophil; CRP, C-reactive protein; sCr, serum creatinine; PCT, procalcitonin; VSD, vacuum sealing drainage.

**Figure 3:**
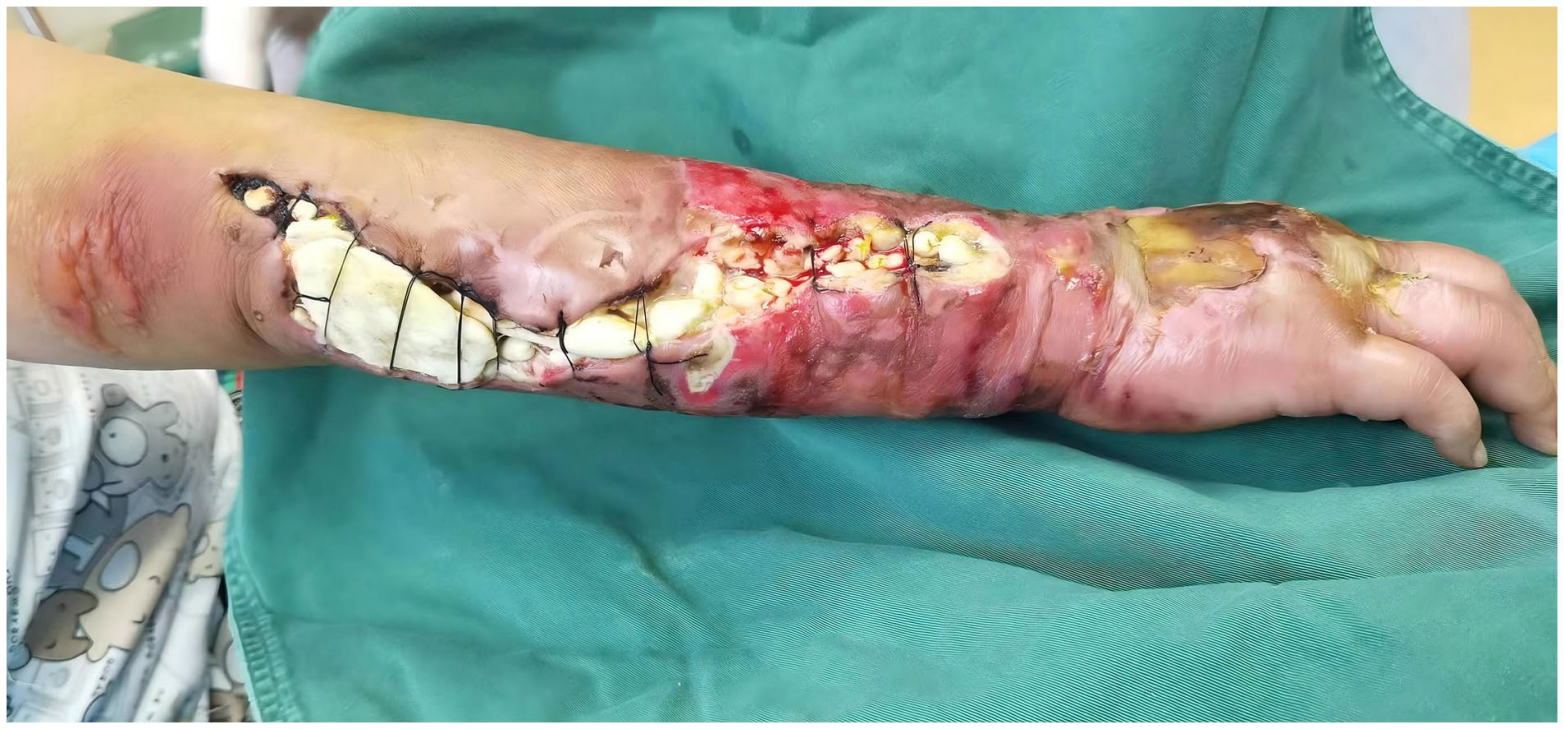
*S. suis* skin and soft tissue abscess. The wound after debridement with implantation of antibiotic-loaded bone cement.

Concurrently with optimization of the antimicrobial regimen, repeat debridement of the infected site was performed. Intraoperative inspection revealed involvement of the subcutaneous tissues and deep fascia, with purulent exudate and fascial necrosis; however, no myonecrosis or bone involvement was identified. Given the extent of the soft-tissue infection, vancomycin-loaded bone cement was placed during the procedure to provide sustained local antibiotic delivery and prevent further spread of infection (Figure 3). After antimicrobial adjustment and repeat debridement, four subsequent blood cultures and wound secretion cultures were all negative for pathogens.

On hospital day 7, she was referred to the orthopedic department for specialized treatment. Following dressing changes and skin grafting, complete wound healing was achieved without any sequelae, including hearing loss—an outcome attributable to the absence of meningeal inflammation and vancomycin-related ototoxicity.

## 3 Discussion

This case report describes a severe infection caused by *S. suis*, an uncommon cause of wound and soft tissue infections. Etiological confirmation enabled appropriate medical and surgical management, including timely adjustment of antimicrobial therapy and surgical debridement combined with vancomycin-loaded bone cement.

*S. suis* is an encapsulated Gram-positive coccus that was first isolated from infected piglets in 1954 (8). Because *S. suis* commonly colonises the upper respiratory tract of pigs—particularly the tonsils and nasal cavities—the respiratory route constitutes the principal mode of transmission between animals. It is also a zoonotic pathogen capable of infecting humans through broken skin or mucous membranes, or via ingestion of undercooked, contaminated pork products. Human infections were first reported in Denmark in 1968, and subsequent cases have been documented worldwide since then. To date, more than 1,000 cases have been reported across over 30 countries, with Southeast Asia identified as a major endemic region, particularly Thailand and Vietnam, due to high pork consumption and local practices of eating raw or undercooked meat (9). In these areas, the gastrointestinal tract is considered the principal route of transmission. Several outbreaks have occurred in rural China, where backyard pig farming and pork consumption are common (10). Notably, two large-scale outbreaks occurred in Jiangsu in 1998 and Sichuan in 2003, resulting in 230 infections and 51 deaths (11). Sporadic cases of *S. suis* infection have been reported over a long period in Guizhou Province, including those caused by serotype 1 (12).

*S. suis* comprises 35 serotypes based on capsular polysaccharide antigens, with serotype 2 accounting for 97% of human infections; serotypes 1, 1/2, and 4 are rare (13). Clinical manifestations depend on the infection site and commonly include meningitis, endocarditis, and epidural abscesses, often accompanied by fever, hypotension, and septic shock. Hearing loss is the most common complication of meningitis, resulting from bacterial virulence factors that dissolve in lymphatic fluid and enter the cochlea via the cochlear aqueduct, causing suppurative labyrinthitis and irreversible damage to cranial nerve VIII (14). In the present case, sepsis and wound infection were the main clinical features. Notably, the patient’s hearing remained unaffected. Given that vancomycin—which was administered in this case—is a potentially ototoxic agent, this favorable outcome was likely attributable to the absence of meningeal involvement, the relatively short duration of therapy (14 days), and the maintenance of drug levels within the recommended therapeutic range.

Virulence is closely linked to genotype, with major virulence factors including mrp, epf, sly, gdh, and fbps (15). Due to resource limitations, genotyping of this isolate was not performed. Nevertheless, on the night of injury, she developed marked wound swelling, which rapidly progressed to septic shock, indicating possible infection with a highly virulent serotype.

*S. suis* is widely distributed in pig farms across Asia, with detection rates exceeding 50% in South Korea and Thailand and generally above 40% in China (16). Furthermore, the carriage rate among healthy pigs in this region has been reported to range from 58.84 to 80% (17).

In addition to occupational exposure, dietary exposure to pork and pork products is another route of *S. suis* transmission to humans. The consumption of raw or undercooked pork and pork-derived products has been associated with both sporadic cases and outbreaks, particularly in Southeast Asia, where traditional dishes prepared with raw pork or pig blood are commonly consumed (18). Immunocompromised conditions have been reported to increase susceptibility to invasive *S. suis* infection, with alcoholism, chronic liver disease, and diabetes mellitus being the most frequently reported predisposing factors (19).

In China, the preparation and cooking of pork typically occur in the kitchen, where utensils for raw and cooked meat are often used interchangeably. This practice may account for cases of gastrointestinal transmission even in the absence of a cultural habit of consuming raw pork. Upon further inquiry, we found that after undergoing debridement, the patient consumed pork broth at a local restaurant—a soup dish in which meat and vegetables are cooked together. Upon further investigation at the local restaurant, we found that utensils used for raw meat and cooked food were washed in the same sink without proper disinfection or separation. One possible scenario is that contamination of the utensils used to serve the pork broth provided the source of infection. If this were the case, *S. suis* could have been introduced through the gastrointestinal tract, and the immune system failed to eradicate the bacteria that translocated from the intestinal lumen into the bloodstream. Similar cases of suspected gastrointestinal transmission of *S. suis* have been reported (20–22). The postoperative wound hematoma may have then provided a favourable milieu for bacterial proliferation and the development of suppurative infection (23). However, this hypothesis remains speculative, and alternative routes of infection cannot be definitively excluded, given that no microbiological testing was performed at the restaurant to confirm the presence of *S. suis*. One such alternative is exogenous contamination during debridement, possibly resulting from non-strict adherence to aseptic technique. In contrast, transmission from the injury environment is highly unlikely, as the patient sustained the wound from a door frame in her urban residence.

Penicillin G has been previously used as a highly effective antimicrobial agent for treating *S. suis* infection. Previous studies have demonstrated that *S. suis* isolates are highly susceptible to penicillin (92–100%), with average MICs ranging from 0.015 to 0.06 μg/mL (19) of note, the isolate from this patient was resistant to penicillin, a finding consistent with surveillance data from this region, which indicate penicillin and tetracycline resistance rates of 21.4 and 92.9%, respectively, among *S. suis* isolates recovered from diseased pigs (24). Some *S. suis* strains carry a range of resistance determinants, including *tet(M), tet(L)* (conferring tetracycline resistance), and *erm(B)* (conferring macrolide resistance) (25). The high prevalence of antimicrobial resistance among *S. suis* strains carried by pigs poses a substantial risk of transmitting resistant bacteria to humans, potentially leading to difficult-to-treat infections. In China, penicillin is a widely recognized antibiotic and can be easily obtained over the counter in pharmacies. This easy access has contributed to the overuse of antibiotics among the general population. Long-term inappropriate antibiotic use in animal husbandry—for infection prevention and growth promotion—has been considered a major driver of antimicrobial resistance in *S. suis*. A survey of livestock farms identified doxycycline, penicillin, and ceftiofur as the three most commonly used veterinary antimicrobials, with usage rates of 81.70, 72.15, and 69.81%, respectively. Such widespread antibiotic use has markedly intensified antimicrobial resistance, a trend similarly observed among *S. suis* isolates from pigs in Thailand. In a 17-year retrospective study of clinical cases conducted at a large tertiary hospital in Thailand (2007–2023), only 48.2% of *S. suis* isolates were fully susceptible to penicillin, while 45.9% exhibited intermediate susceptibility and 5.9% were classified as penicillin-resistant. Notably, penicillin minimum inhibitory concentrations (MICs) increased significantly over the study period (*p* < 0.001, 19). On reviewing the clinical course in this case, empirical piperacillin-tazobactam therapy was initiated at admission. However, clinical improvement was not observed until the antimicrobial regimen was revised to vancomycin, guided by culture findings from both blood and wound exudates and their susceptibility profiles. Given increasing penicillin non-susceptibility, non-*β*-lactam agents such as vancomycin and fluoroquinolones may serve as alternative treatment options. For patients presenting with skin and soft-tissue infections, the combination of intravenous and topical antibiotic administration also represents a feasible approach.

Although no myonecrosis or bone involvement was observed, the extensive involvement of the subcutaneous tissues and deep fascia posed a high risk of persistent infection despite systemic antibiotic therapy, warranting the placement of vancomycin-impregnated bone cement. Sustained local antibiotic release from bone cement beads achieves supratherapeutic concentrations at the infection site without significant systemic toxicity, an approach that is particularly advantageous in poorly vascularized or necrotic fascial tissues (26, 27). Clinical studies have further demonstrated that vancomycin-containing bone cement promotes soft-tissue healing and reduces infection recurrence in patients with bone infections and soft-tissue defects (28, 29). Meanwhile, adjunctive agents that reduce inflammatory mediator levels, such as Xuebijing and ulinastatin, may also be considered, as in the present case. Xuebijing is an injectable traditional Chinese medicine composed of five herbal ingredients: *Carthami Flos, Paeoniae Radix Rubra, Chuanxiong Rhizoma, Salviae Miltiorrhizae Radix et Rhizoma, and Angelicae Sinensis Radix*. Ulinastatin, also known as urinary trypsin inhibitor, is a naturally occurring serine protease inhibitor with anti-inflammatory and immunomodulatory properties. A recent umbrella review of meta-analyses concluded that the combination of ulinastatin and Xuebijing is associated with improved clinical outcomes—including reduced 28-day mortality, shorter intensive care unit stay, and shorter duration of mechanical ventilation—likely mediated by immunomodulation, inhibition of inflammatory mediator production and release, and protection of mitochondrial and endothelial function (30).

Based on this case, we summarised the key clinical features of these wound infections, including rapid onset after exposure, pale yellow and thin purulent discharge, and a tendency for local spread, often accompanied by severe systemic complications such as streptococcal toxic shock syndrome or meningitis.

## 4 Strengths and limitations

This case report provides a complete record of the entire treatment process for a critically ill patient with wound infection and sepsis caused by *S. suis*. The clinical data are comprehensive and detailed, including a precise intraoperative description of the infected tissues (subcutaneous tissues and deep fascia with purulent exudate and fascial necrosis, without myonecrosis or bone involvement). We confirmed the pathogen through both wound secretion culture and blood cultures. At the same time, we implemented a surgical strategy involving vancomycin-loaded bone cement implantation after debridement, which effectively controlled the infection and enabled successful subsequent skin grafting. This approach provides a valuable practical reference for the management of similar severe soft tissue and systemic infections. However, this study has several limitations. First, the results are based on a single patient and thus have limited generalizability. Second, the proposed gastrointestinal transmission route remains speculative, and alternative sources of infection cannot be definitively excluded. Third, molecular confirmation of the *S. suis* isolate was not performed, raising the possibility of misidentification with other streptococcal species.

## 5 Conclusion

This report highlights an exceedingly rare presentation of extensive wound infection due to *S. suis*. We report this case to highlight the importance of clinicians considering a broad range of potential sources when evaluating wound infections. Potential routes of acquisition—including direct wound contamination, hematogenous spread from distant sites, and foodborne transmission—should all be considered during clinical evaluation. This case also underscores crucial public health concerns, particularly the potential risk of *S. suis* transmission through contaminated or inadequately cooked pork products, a notable issue in regions such as China, where pig farming and pork consumption are widespread. Raising awareness of this condition among clinicians and laboratory staff is crucial for minimizing misdiagnoses. Ultimately, strengthening food-safety oversight and ensuring proper handling and preparation of pork remain critical measures for preventing such infections at the population level.

### 5.1 Patient perspective

After achieving infection control with intravenous vancomycin combined with metronidazole, along with surgical debridement and implantation of vancomycin-loaded bone cement, the patient’s clinical condition improved progressively. Reflecting on her treatment experience, the patient reported that, in the early days after admission, she experienced dizziness, generalized weakness, and severe wound pain. Despite undergoing debridement, the wound swelling continued to worsen. She noted that significant relief was achieved only after antibiotic-loaded bone cement was applied, after which the pain gradually subsided and her overall condition improved day by day.

At the time of discharge, the patient stated that the wound had essentially healed, forearm flexion and extension had returned to normal, and she felt in excellent condition. At the 1-year telephone follow-up, the patient reported no residual discomfort and indicated that her quality of life had not been affected.

## Data Availability

The original contributions presented in the study are included in the article/supplementary material, further inquiries can be directed to the corresponding author.
